# Porcine Deltacoronavirus Accessory Protein NS7a Antagonizes IFN-β Production by Competing With TRAF3 and IRF3 for Binding to IKKε

**DOI:** 10.3389/fcimb.2020.00257

**Published:** 2020-06-12

**Authors:** Puxian Fang, Liurong Fang, Sijin Xia, Jie Ren, Jiansong Zhang, Dongcheng Bai, Yanrong Zhou, Guiqing Peng, Shuhong Zhao, Shaobo Xiao

**Affiliations:** ^1^State Key Laboratory of Agricultural Microbiology, College of Veterinary Medicine, Huazhong Agricultural University, Wuhan, China; ^2^Key Laboratory of Preventive Veterinary Medicine in Hubei Province, The Cooperative Innovation Center for Sustainable Pig Production, Wuhan, China; ^3^Key Laboratory of Agricultural Animal Genetics, Breeding, and Reproduction of Ministry of Education, College of Animal Science and Veterinary Medicine, Huazhong Agricultural University, Wuhan, China

**Keywords:** porcine deltacoronavirus, accessory protein, NS7a, immune evasion, interferon

## Abstract

As an emerging swine enteropathogenic coronavirus, porcine deltacoronavirus (PDCoV) not only causes serious diarrhea in suckling piglets but also possesses the potential for cross-species transmission, which has sparked growing interest when studying this emerging virus. We previously identified a novel accessory protein NS7a encoded by PDCoV; however, the function of NS7a was not resolved. In this study, we demonstrated that PDCoV NS7a is an interferon antagonist. Overexpression of NS7a notably inhibited Sendai virus (SeV)-induced interferon-β (IFN-β) production and the activation of IRF3 rather than NF-κB. NS7a also inhibited IFN-β promoter activity induced by RIG-I, MDA5, MAVS, TBK1, and IKKε, which are key components of the RIG-I-like receptor (RLR) signaling pathway but not IRF3, the transcription factor downstream of TBK1/IKKε. Surprisingly, NS7a specifically interacts with IKKε but not with the closely related TBK1. Furthermore, NS7a interacts simultaneously with the kinase domain (KD) and the scaffold dimerization domain (SDD) of IKKε, competing with TRAF3, and IRF3 for binding to IKKε, leading to the reduction of RLR-mediated IFN-β production. The interactions of TRAF3-IKKε and IKKε-IRF3 are also attenuated in PDCoV-infected cells. Taken together, our results demonstrate that PDCoV NS7a inhibits IFN-β production by disrupting the association of IKKε with both TRAF3 and IRF3, revealing a new mechanism utilized by a PDCoV accessory protein to evade the host antiviral innate immune response.

## Introduction

Porcine deltacoronavirus (PDCoV), a newly emerging swine enteropathogenic coronavirus, belongs to the genus Deltacoronavirus in the family *Coronaviridae* of the order *Nidovirales* (Wang et al., 2019). PDCoV was first detected in Hong Kong in 2012 (Woo et al., 2012), and this was followed by outbreaks in multiple states of the United States in 2014 (Wang et al., 2014a,b). Subsequently, PDCoV was discovered in other countries, including South Korea (Lee and Lee, 2014; Jang et al., 2018), mainland China (Dong et al., 2015; Song et al., 2015; Wang Y. W. et al., 2015), Thailand, Lao People's Democratic Republic, Vietnam (Janetanakit et al., 2016; Saeng-Chuto et al., 2017), and Japan (Suzuki et al., 2018). PDCoV infection causes typical clinical symptoms characterized by acute diarrhea and vomiting—even mortality—in piglets, leading to economic losses for the swine industry (Jung et al., 2015; Ma et al., 2015; Zhang, 2016). In addition, recent studies have demonstrated that chicken and calves are also susceptible to PDCoV infection (Jung et al., 2017; Liang et al., 2019) and that PDCoV possesses the potential to infect humans (Li et al., 2018), which has sparked growing interest in studying this emerging coronavirus.

Interferon (IFN) and the IFN-induced cellular antiviral response are important components of the innate immune response that constitutes the first line of defense against viral infection (Randall and Goodbourn, 2008). RNA virus infection produces a double-strand RNA (dsRNA) replication intermediate, which represents pathogen-associated molecular patterns (PAMPs). Upon binding to cytoplasmic viral PAMPs, host pattern-recognition receptors in the cytoplasm, such as retinoic acid-induced gene I (RIG-I) and melanoma differentiation gene 5 (MDA5), are activated, facilitating the aggregation of mitochondrial signaling adapter (MAVS), and recruitment of TANK binding kinase 1 (TBK1)/I-kappa B kinase ε (IKKε) (Kawai and Akira, 2009; Belgnaoui et al., 2011). This event leads to the activation of transcription factors interferon regulation factor 3 (IRF3) and nuclear factor κB (NF-κB) and subsequent production of type I IFNs (Fitzgerald et al., 2003; Fang R. et al., 2017). The secreted type I IFNs bind to receptors, leading to the activation of the Janus kinase (JAK)/signal transducers, and activators of transcription (STAT) signaling pathway and the production of hundreds of IFN-stimulated genes (Stark et al., 1998). Due to the deleterious effects of this response on viral replication, many viruses, including coronaviruses (CoVs), have developed various strategies to counteract IFN production and signaling transduction. Some CoVs, including porcine epidemic diarrhea virus (PEDV), severe acute respiratory syndrome coronavirus (SARS-CoV), Middle East respiratory syndrome coronavirus (MERS-CoV), and mouse hepatitis virus (MHV), antagonize IFN production. Detailed inhibitory mechanisms have been revealed and multiple viral proteins involved in the inhibition process have been identified (Vijay and Perlman, 2016; Zhang and Yoo, 2016; Deng et al., 2017; Case et al., 2018). As an emerging CoV, PDCoV has also been reported to suppress type I IFN production (Luo et al., 2016). However, the molecular mechanisms used by PDCoV to antagonize IFN production remain largely unknown.

Accessory proteins are unique proteins encoded by CoVs; however, their number and sequence vary amongst the numerous species of CoVs. For example, SARS-CoV encodes the largest number of accessory proteins, containing ORFs 3a, 3b, 6, 7a, 7b, 8a, 8b, and 9b, whereas only one accessory protein, ORF3, has been identified in PEDV (Liu et al., 2014). Accessory proteins of CoVs are generally not essential for normal viral replication *in vitro* (Tan et al., 2006). However, extensive evidence indicates that many accessory proteins are closely associated with viral pathogenicity, optimal replication, and immune regulation, especially in relation to the regulation of IFN signaling. For example, at least four accessory proteins (ORFs 3b, 6, 8b, and 9b) encoded by SARS-CoV were identified as type I IFN antagonist (Frieman et al., 2007; Liu et al., 2014; Wong et al., 2018). MERS-CoV ORF4a and ORF4b, MHV accessory protein ns2, transmissible gastroenteritis virus (TGEV) protein 7, and feline infectious peritonitis virus (FIPV) protein 7a have also been demonstrated to inhibit IFN responses (Zhao et al., 2012; Cruz et al., 2013; Niemeyer et al., 2013; Dedeurwaerder et al., 2014; Comar et al., 2019). PDCoV encodes at least three accessory proteins, NS6, NS7, and NS7a (Fang et al., 2016; Fang P. et al., 2017). PDCoV NS6 inhibits IFN-β production by interacting with RIG-I and MDA5 to attenuate their association with double-stranded RNA (Fang et al., 2018). Currently, the role of other accessory proteins encoded by PDCoV in the regulation of the IFN signaling pathway remains unclear.

In this study, we demonstrated that PDCoV NS7a also antagonizes IFN-β production *in vitro*. Mechanistically, PDCoV NS7a specifically targets the key kinase IKKε to disrupt its interaction with both TRAF3 and IRF3, thereby blocking the production of IFN-β.

## Materials and Methods

### Viruses, Cells, and Reagents

PDCoV strain CHN-HN-2014 (GenBank accession number KT336560) was isolated in China in 2014 from a piglet with severe diarrhea (Dong et al., 2016). Vesicular stomatitis virus-expressing green fluorescent protein (VSV-GFP) was generously gifted by Dr. Zhigao Bu at Harbin Veterinary Research Institute, Chinese Academy of Agricultural Sciences. Sendai virus (SeV) was obtained from the Center of Virus Resource and Information, Wuhan Institute of Virology, Chinese Academy of Sciences. Human embryonic kidney cells (HEK-293T), and porcine intestinal epithelial cells (IPI-2I) were obtained from the China Center for Type Culture Collection. Porcine kidney epithelial cells (LLC-PK1) were obtained from American Type Culture Collection. They were cultured and maintained at 37°C and 5% CO_2_ in Dulbecco's Modified Eagle's medium (Invitrogen, NY, USA) supplemented with 10% heat-inactivated fetal bovine serum (FBS) (PAN-biotech, Bavaria, Germany). Antibodies against p-IRF3, p65, Lamin A/C, and β-actin were purchased from ABclonal (Wuhan, China). Rabbit anti-p-p65, anti-IRF3, and TNF receptor-associated factor 3 (TRAF3) were purchased from Cell Signaling Technology (Danvers, MA, USA). Mouse anti-Flag, anti-GFP, -Myc, or -hemagglutinin (HA) antibodies were purchased from Medical and Biological Laboratories (Nagoya, Japan). Mouse anti-Hsp90 were purchased from BD Transduction Labs (NJ, NY, USA). Horseradish peroxidase (HRP)-conjugated secondary antibodies and 4′, 6-diamidino-2-phenylindole (DAPI) were purchased from Beyotime (Shanghai, China). Goat anti-mouse IgG LCS/HCS were purchased from Abbkine (California, USA). Alexa Fluor 594-conjugated donkey anti-mouse IgG and Alexa Fluor 488-conjugated donkey anti-rabbit IgG were obtained from Jackson ImmunoResearch (PA, USA). NE-PER nuclear and cytoplasmic extraction reagents kit was purchased from Thermo Fisher Scientific (IL, USA).

### Plasmids and Dual-Luciferase Reporter Assay

The NS7a gene from the PDCoV strain CHN-HN-2014 was amplified using primers NS7a-F and NS7a-R (Table 1) and then cloned into pCAGGS-Myc-N with an N-terminal Myc tag to yield pCAGGS-Myc-NS7a. Expression plasmid pCAGGS-HA-NS7a was constructed as described previously (Fang P. et al., 2017). Flag-tagged expression constructs encoding RIG-I, MDA5, MAVS, TBK1, IKKε, and IRF3 have been described previously (Ding et al., 2014; Fang et al., 2018). The HA-tagged expression construct encoding IKKε, pCAGGS-HA-IKKε, was constructed by cloning the full-length cDNA of IKKε into the pCAGGS-HA-N vector with primers IKKε-F and IKKε-R (Table 1). The Flag-tagged full-length IKKε expression plasmid was used as the template to amplify two functional domains of IKKε, the kinase domain (KD) (IKKε aa 1–304) and scaffold dimerization domain (SDD) (IKKε aa 383–648), as reported previously (Fang R. et al., 2017), which were cloned into the pCAGGS-Flag/HA-N vector with an N-terminal Flag/HA tag using specific primers (Table 1). The resulting expression plasmids were named pCAGGS-Flag/HA-KD and pCAGGS-Flag/HA-SDD. All expression constructs were validated via DNA sequencing. For the luciferase reporter assay, cells cultured in 24-well plates were co-transfected with a luciferase reporter plasmid (IFN-β-Luc, NF-κB-Luc, or IRF3-Luc) and pRL-TK (Promega, WI, USA), along with the indicated plasmids or empty vector for 24 h, and were then infected with SeV (10 hemagglutinating activity units/well). At 12 h post-infection, the cells were lysed, and firefly luciferase and Renilla luciferase activities were detected through the dual-luciferase reporter assay system according to the protocol from the manufacturer (Promega). Representative data from three independently conducted experiments are shown as the relative firefly luciferase activities with normalization to the Renilla luciferase activities.

**Table 1:** Primers used for the construction of plasmids and real-time RT-PCR assay.

**Primer**	**Nucleotide sequence (5′- 3′)**
NS7a-F	GCTGAATTCATGGCCCAGCTCAAGGTTTC
NS7a-R	TCACTCGAGCTAGAGCCATGATGCGAGGA
IKKε-F	TGGGAATTCATGCAGAGCACAGCCAATT
IKKε-R	TGTGGTACCTCAGACATCAGGAGGTGCT
SDD-F	TTGGAATTCATGAGCACAGCCATCCCTAAG
SDD-R	TATGGTACCTCACTCTTCCAGGAGCTTGCTGAG
KD-F	TGGGAATTCATGCAGAGCACAGCCAATT
KD-R	ATAGGTACCTCAACTGGTCTCCGCAAAGAACT
h-IFN-β-F	TCTTTCCATGAGCTACAACTTGCT
h-IFN-β-R	GCAGTATTCAAGCCTCCCATTC
h-GAPDH-F	TCATGACCACAGTCCATGCC
h-GAPDH-R	GGATGACCTTGCCCACAGCC

### RNA Extraction and Quantitative Real-Time RT-PCR

HEK-293T cells in 24-well plates were transfected with increasing amounts of NS7a expression plasmids or empty vector. After 24 h, the cells were left untreated or infected with SeV for 12 h. Total RNA was extracted from the cells using TRIzol reagent (Invitrogen), followed by first-strand cDNA synthesis by using avian myeloblastosis virus (AMV) reverse transcriptase (TaKaRa, Japan) with the indicated primers (Table 1). The above cDNA (0.5 μl of the 20 μl RT reaction mixture) were used as templates and subjected to SYBR green PCR assays (Applied Biosystems) at least three times. The results are expressed as the relative gene expression level with normalization to the expression level of glyceraldehyde-3-phosphate dehydrogenase (GAPDH).

### Lentivirus Packaging

The lentiviral expression plasmid pLvx-P2A-mCherry-IKKε-Flag was generated by cloning IKKε with the Flag tag at the C-terminus into the lentiviral vector pLvx-P2A-mCherry, which was constructed via the introduction of the P2A sequence (GGAAGCGGAGCTACTAACTTCAGCCTGCTGAAGCAGGCTGGAGACGTGGAGGAGAACCCTGGACCT) into the N-terminal of mCherry in the lentiviral vector pLvx-mCherry (TaKaRa, Japan), as reported previously (Wang Y. et al., 2015). Lentivirus-assisted plasmids pLP1, pLP2, and pLP-VSV-G were kindly provided by Dr. Xing Liu at the Institute of Veterinary Medicine, Jiangsu Academy of Agricultural Sciences. The recombinant lentiviruses were packaged as described previously (Ke et al., 2017). For the establishment of a cell line overexpressing IKKε-Flag, IPI-2I cells cultured in 24-well plates were inoculated with the recombinant lentivirus expressing IKKε-Flag. After 24 h, the cells were passaged and 1 μg/ml of puromycin (Sigma, MA, USA) was added to the cell culture for positive cell selection. The positive cells were further cloned by the limited dilution method continuously and the obtained cell clones overexpressing IKKε-Flag were confirmed by fluorescence microscopy and a western blot assay.

### IFN Bioassay

IFN bioassays were performed in HEK-293T cells by using recombinant VSV-GFP as described previously (Cardenas et al., 2006; Fang et al., 2018).

### Co-immunoprecipitation (Co-IP) and Western Blot Analysis

For the Co-IP experiment, cells grown in 60-mm dishes were co-transfected with the appropriate expression constructs encoding Flag, HA, or Myc-tagged proteins for 24 h. The cells were washed with phosphate-buffered saline (PBS) and then lysed for 40 min at 4°C with 0.5 ml of lysis buffer (50 mM Tris-HCl, 150 mM NaCl, 1% NP-40, 10% glycerin, 0.1% SDS, 2 mM Na_2_EDTA, pH 7.4). A portion of each supernatant from the lysed cells was used in the whole-cell extract assay. The remaining portions of the supernatants were immunoprecipitated with indicated antibodies overnight at 4°C, followed by the addition with protein A+G agarose beads (Beyotime, Shanghai, China) for 4 h at 4°C. The agarose beads containing immunocomplexes were washed three times with 1 ml of lysis buffer. Whole-cell lysates (WCL) and immunoprecipitation (IP) complexes were separated by 12% SDS-PAGE and transferred to a polyvinylidene difluoride membrane (Millipore, Darmstadt, Germany). The membrane containing proteins were blocked with 5% nonfat milk in PBST with 0.1% polysorbate-20, followed by treatment with the indicated primary antibodies. Four hours later at room temperature, the membranes were washed three times with PBST, and then incubated with HRP-conjugated secondary antibodies (Beyotime, Shanghai, China). After 45 min at room temperature, the membrane was washed three times and then visualized via enhanced chemiluminescence reagents (Bio-Rad, California, USA). β-actin served as a protein loading control and was detected with a mouse anti-β-actin monoclonal antibody.

### Indirect Immunofluorescence Assay (IFA)

The IFA was performed as described previously (Fang et al., 2018). Briefly, cells seeded onto coverslips in 24-well plates were treated according to various experiments. At the indicted time, the cells were harvested and fixed with 4% paraformaldehyde for 15 min and then permeated with methyl alcohol for 10 min at room temperature. The cells were washed with PBST and then blocked with 5% bovine serum albumin for 1 h at 37°C. The cells were incubated separately with primary antibodies for 1 h at 37°C followed by treatment with Alexa Fluor 594-conjugated donkey anti-mouse IgG and Alexa Fluor 488-conjugated donkey anti-rabbit IgG as secondary antibodies for 1 h at 37°C. Subsequently, the above treated cells were stained with DAPI for 15 min at room temperature. Fluorescent images were visualized by using a confocal laser scanning microscope (Fluoviewver.3.1; Olympus, Japan) after three washes with PBS.

### Statistical Analysis

Statistical differences were determined by one-way ANOVAs using GraphPad Prism 5.0 software. For all experiments, differences were considered to be statistically significant when *p*-values were < 0.05.

## Results

### PDCoV NS7a Impairs SeV-Induced IFN-β Production

**Figure 1:**
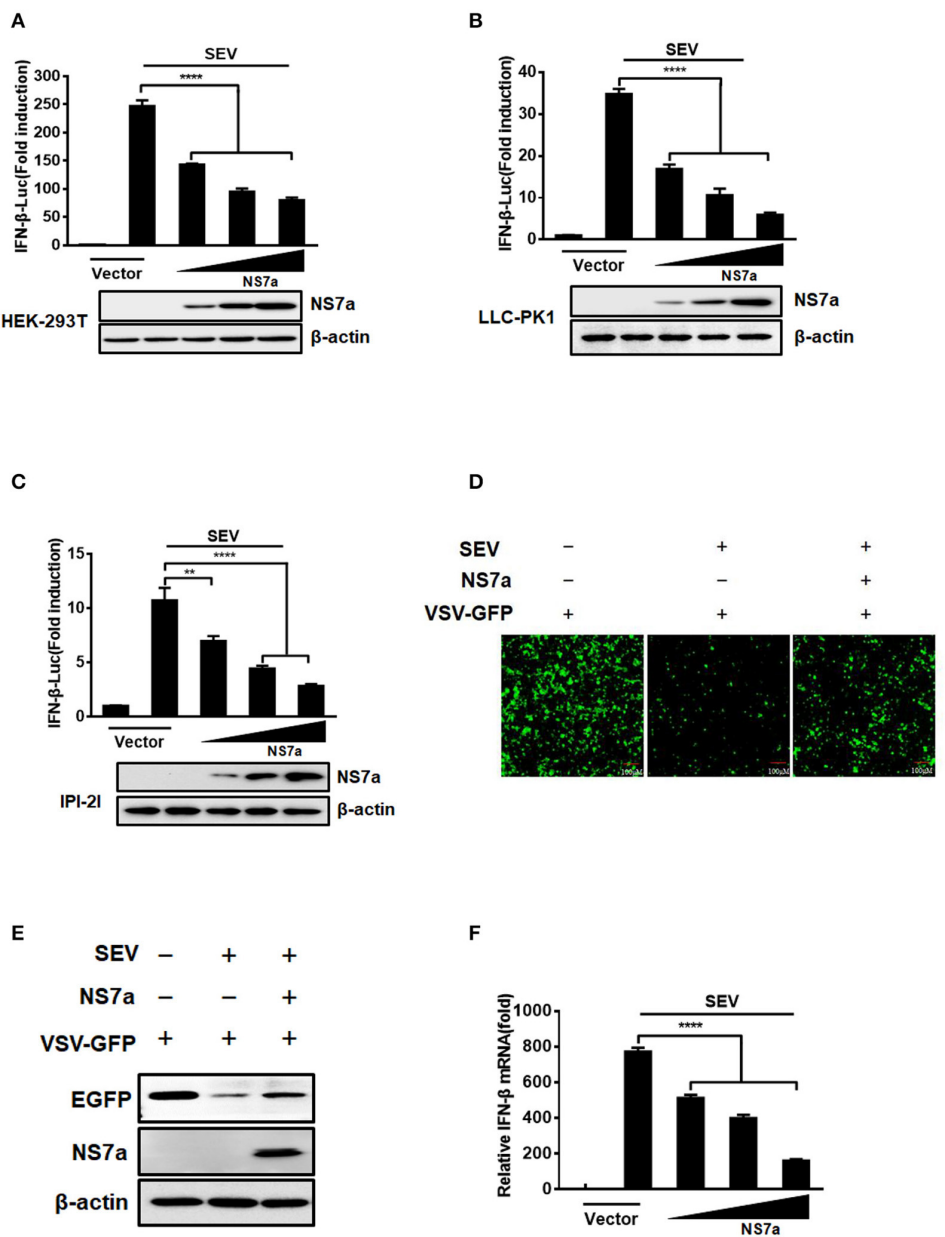
NS7a inhibits SeV-induced IFN-β production. **(A)** HEK-293T cells, **(B)** LLC-PK1 cells, or **(C)** IPI-2I cells were co-transfected with IFN-β-Luc and pRL-TK plasmids, along with increasing quantities of the pCAGGS-HA-NS7a plasmid for 24 h. This was followed by treatment with or without SeV (10 hemagglutination activity units/well) for 12 h. The cells were lysed and subjected to the dual-luciferase assay. The expression of NS7a protein and β-actin were detected via a western blot assay with antibodies against HA and β-actin, respectively. β-actin served as a protein loading control. **(D,E)** HEK-293T cells were transfected with the indicated amount of the pCAGGS-HA-NS7a plasmid or empty vector for 24 h and then infected with SeV for 12 h. The cell supernatants were collected and subjected to UV irradiation treatment, followed by addition to the monolayer of HEK-293T cells in a 24-well plate for 24 h. The cells were inoculated with VSV-GFP for 12 h, and this was followed by the observation of viral replication via fluorescence microscopy **(D)** and western blot assay **(E)**. **(F)** HEK-293T cells were transfected with increasing amounts of the pCAGGS-HA-NS7a plasmid for 24 h, and this was followed by treatment with or without SeV. At 12 h after infection, the cells were collected, and total RNA was extracted to detect the expression level of IFN-β and GAPDH by SYBR Green PCR assay. The results represent data from three independent experiments, **p < 0.01; ****p < 0.0001.

To determine whether PDCoV NS7a inhibits IFN-β production, HEK-293T cells, LLC-PK1 cells, or IPI-2I were co-transfected with increasing amounts of pCAGGS-HA-NS7a or empty vector along with the firefly luciferase reporter plasmid IFN-β-Luc and *Renilla* luciferase reporter plasmid pRL-TK (as an internal control) for 24 h, and this was followed by treatment with SeV for 12 h. The cell lysates were collected and subjected to the dual-luciferase reporter assay. The results showed that SeV significantly induced the activation of the IFN-β-Luc promoter, but upregulation of IFN-β promoter activation was impaired by NS7a protein expression in all three tested cell lines (Figures 1A–C, Supplementary Tables 1–3). Additionally, an IFN bioassay was performed using an IFN-sensitive VSV-GFP in HEK-293T cells. Consistent with the results from the IFN-β-Luc reporter assay, cellular supernatants from SeV-infected cells notably restricted the replication of VSV-GFP (Figure 1D). However, the natural replication of VSV-GFP was, to a large extent, restored via the presence of supernatants from cells expressing NS7a when compared with that of supernatants from empty vector-transfected cells. In line with the results from Figure 1D, western blot results showed that the expression of EGFP was significantly inhibited by cellular supernatants from SeV-infected cells but was partially rescued in the presence of supernatants from cells expressing NS7a (Figure 1E). To confirm the inhibition of IFN-β production on the mRNA level by PDCoV NS7a, the real-time RT-PCR assay was performed in HEK-293T cells. As shown in Figure 1F, ectopic expression of NS7a notably inhibited SeV-induced IFN-β mRNA expression levels. These results strongly indicate that PDCoV NS7a antagonizes IFN-β production.

### PDCoV NS7a Inhibits Activation of IRF3 but Not NF-κB

**Figure 2:**
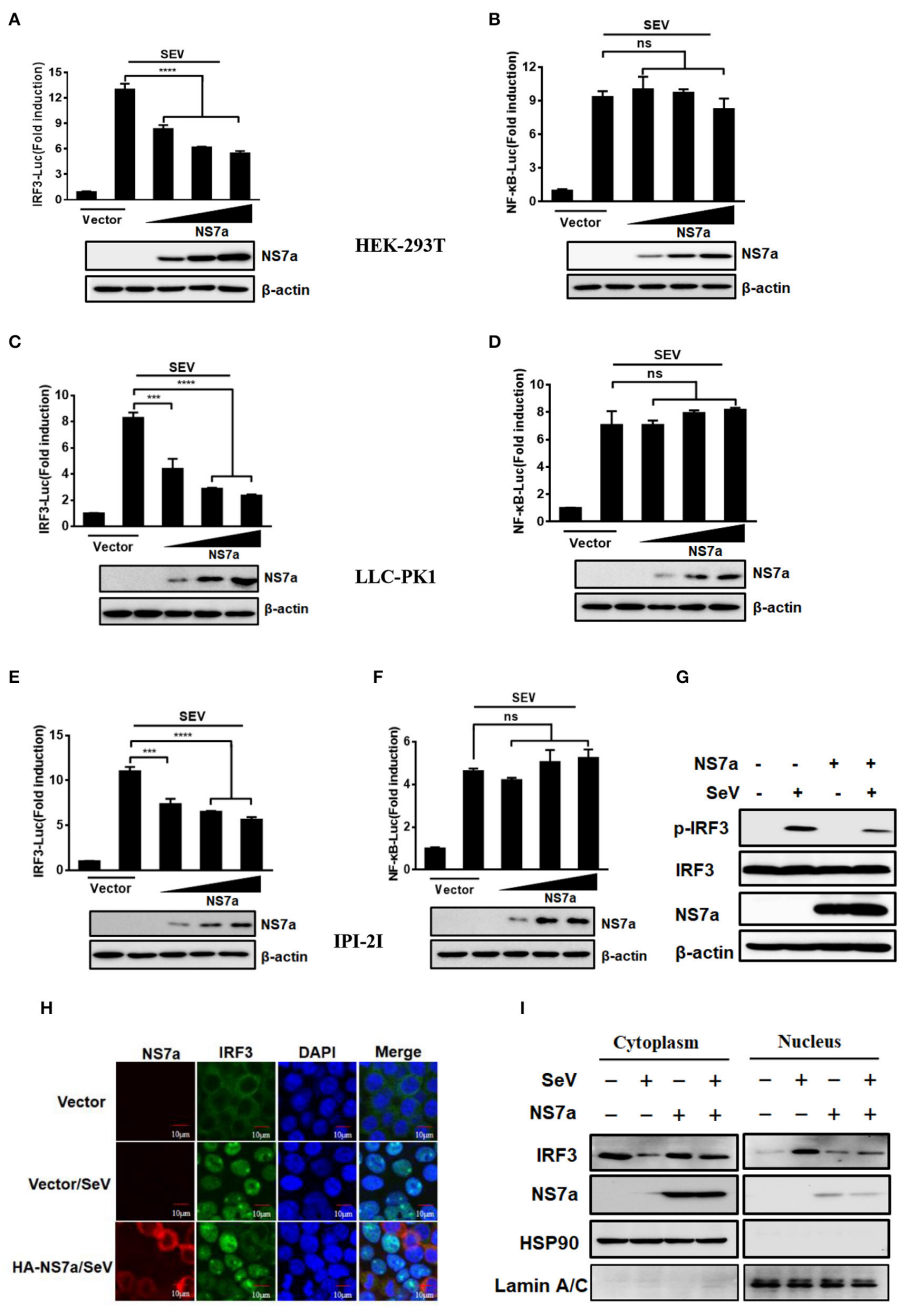
NS7a impedes activation of IRF3 but not NF-κB. **(A,B)** HEK-293T cells, **(C,D)** LLC-PK1 cells, or **(E,F)** IPI-2I cells were co-transfected with IRF3-Luc **(A,C,E)**, or NF-κB-Luc **(B,D,F)**, and pRL-TK plasmids together with the indicated amount of the HA-NS7a expression plasmid or empty vector for 24 h. This was followed by infection with SeV for 12 h and subsequently the dual-luciferase assay. Antibodies against HA and β-actin were used to detected expression of NS7a and β-actin via a western blot assay, respectively. β-actin acted as a protein loading control. ***p < 0.001; ****p < 0.0001. Non-significant differences in data are expressed as “ns.” **(G)** HEK-293T cells were transfected with the pCAGGS-HA-NS7a or empty vector for 24 h and then infected with SeV or left untreated. At 8 h after infection, the cells were lysed and subjected to the western blot assay with primary antibodies against phosphorylated IRF3 (p-IRF3 Ser386) and total IRF3, HA, and β-actin, respectively. **(H)** HEK-293T cells were transfected with the pCAGSS-HA-NS7a or empty vector for 24 h and then infected with SeV or left untreated for 8 h as described for **(G)**. The cells were fixed and subjected to an immunofluorescence assay with rabbit anti-IRF3 and mouse anti-HA used as primary antibodies, followed by treatment with secondary antibodies Alexa Fluor 594-conjugated donkey anti-mouse IgG (red) or Alexa Fluor 488-conjugated donkey anti-rabbit IgG (green). The cell nuclei were stained with DAPI (blue). Fluorescent images were acquired with a confocal laser scanning microscope (Fluoview ver. 3.1; Olympus, Japan). **(I)** HEK-293T cells were transfected with the pCAGSS-HA-NS7a or empty vector for 24 h and then infected with SeV or left untreated for 8 h as described for **(H)**, followed by western blot analysis of IRF3 in nuclear and cytoplasmic fractions. Hsp90 served as cytoplasmic control. Lamin A/C served as nuclear protein control.

Two key transcription factors IRF3 and NF-κB that bind to distinct regulatory domains in the IFN-β promoter are essential for the induction of IFN-β production. Since PDCoV NS7a inhibits IFN-β production, we further investigated the effect of NS7a on SeV-induced activation of IRF3 and NF-κB. To this end, HEK-293T cells, LLC-PK1 cells, or IPI-2I cells were co-transfected with the NS7a expression plasmid and reporter plasmid IRF3-Luc or NF-κB-Luc along with the internal control plasmid pRL-TK for 24 h, and this was followed by stimulation with SeV for 12 h. As shown in Figure 2, SeV infection induced promoter activities of IRF3 and NF-κB significantly, whereas overexpression of NS7a remarkably impaired the SeV-induced promoter activities of IRF3 (Figures 2A,C,E, Supplementary Tables 4, 6, 8) in a dose-dependent manner; however, NS7a did not inhibit NF-κB promoter activation (Figures 2B,D,F, Supplementary Tables 5, 7, 9).

IRF3 is a key transcription factor for the induction of type I IFN, which is activated via phosphorylation and nuclear translocation, followed by the coordinated assembly of activated transcription factors and its binding to the promoter of specific defense genes to improve their transcription in the cell nucleus (Sato et al., 1998). Because NS7a blocks SeV-induced IRF3-dependent promoter activity, the effect of NS7a on the phosphorylation and nuclear translocation of IRF3 was further investigated via western blotting and IFA. As expected, SeV infection significantly upregulated the level of IRF3 phosphorylation when compared with that of the mock-treated cells; however, the increase was inhibited by NS7a markedly (Figure 2G). Consistent with the western blot results, the nuclear translocation of IRF3 was also blocked by NS7a (Figure 2H). To further verify the results from IFA in Figure 2H, we performed nuclear and cytoplasmic fractionation of the cells following SeV treatment according to the manufacturer's protocol. As expected, higher levels of IRF3 were found in the nuclear fraction than in the cytoplasmic fraction after SeV treatment of vector-transfected cells (Figure 2I, lanes 2 and 6). In contrast, in the NS7a-transfected cells after SeV stimulation, more IRF3 proteins were detected in the cytoplasmic fraction than in the nuclear fraction (Figure 2I, lanes 4 and 8). Taken together, these results strongly support the notion that PDCoV NS7a is an IFN antagonist that impairs the activation of IRF3.

### NS7a Disrupts IFN-β Promoter Activation Driven by RIG-I, MDA5, MAVS, TBK1, and IKKε but Not IRF3

**Figure 3:**
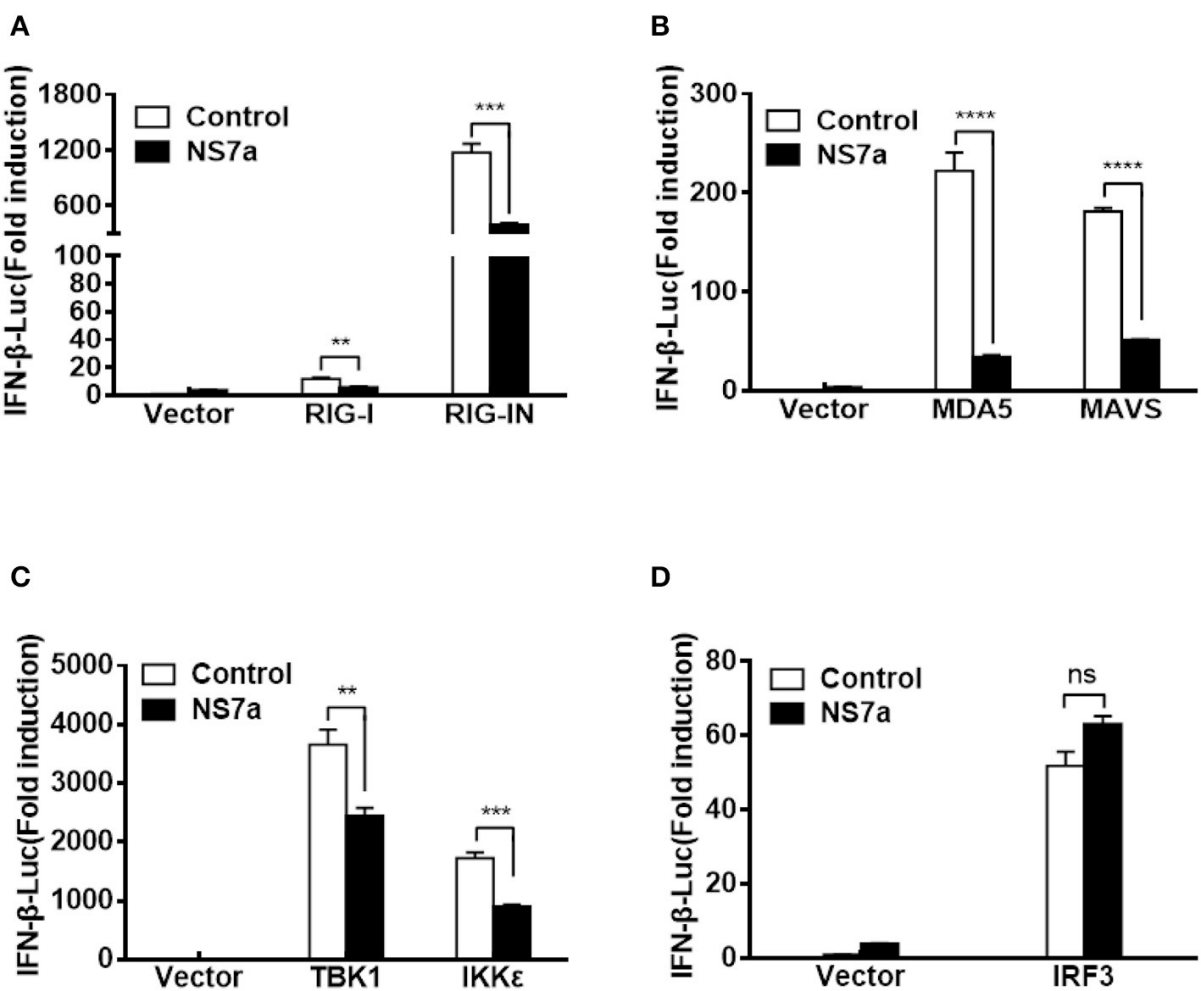
NS7a disrupts TBK1/IKKε-mediated IFN-β production but not that induced by IRF3. HEK-293T cells were co-transfected with pCAGGS-HA-NS7a and Flag-tagged RIG-I/RIG-IN **(A)**, MDA5, MAVS **(B)**, TBK1, IKKε **(C)**, or IRF3 **(D)** expression plasmids together with IFN-β-Luc and pRL-TK for 24 h. The cells were lysed and subjected to dual-luciferase assays. The results presented are representative of the means and standard deviations of data from three independent experiments. The relative firefly luciferase activity was relative to that of an untreated empty vector control with normalization to the Renilla reniformis luciferase activity. **p < 0.01; ***p < 0.001; ****p < 0.0001; Non-significant differences in data are expressed as “ns”.

Because NS7a inhibits the SeV-induced activation of IRF3 and IFN-β production, and SeV is a strong inducer of the RIG-I-like receptor (RLR)-mediated IFN signaling pathway (Yoshida et al., 2018), we hypothesized that NS7a targets one or several molecules among the RLR pathway to inhibit IFN-β production. To test this hypothesis, HEK-293T cells were co-transfected with pCAGGS-HA-NS7a and a series of expression plasmids encoding the key molecules of the RLR signaling pathway, including RIG-I, RIG-IN (a constitutively activated RIG-I mutant), MDA-5, MAVS, TBK1, IKKε, and IRF3 along with the IFN-β-Luc reporter plasmid and pRL-TK. The results of the dual-luciferase reporter assay indicated that all tested molecules of the RLR signaling pathway induced a significant activation of the IFN-β promoter (Figures 3A–D, Supplementary Tables 10, 11). However, RIG-I/RIG-IN-, MDA-5-, MAVS-, TBK1-, and IKKε-mediated activation of the IFN-β promoter was notably blocked by the expression of NS7a. In contrast, NS7a did not inhibit IRF3-mediated IFN-β promoter activation (Figure 3D). These results suggested that NS7a targets the RLR signaling pathway at the level of TBK1/IKKε.

### NS7a Interacts With IKKε but Not TBK1

**Figure 4:**
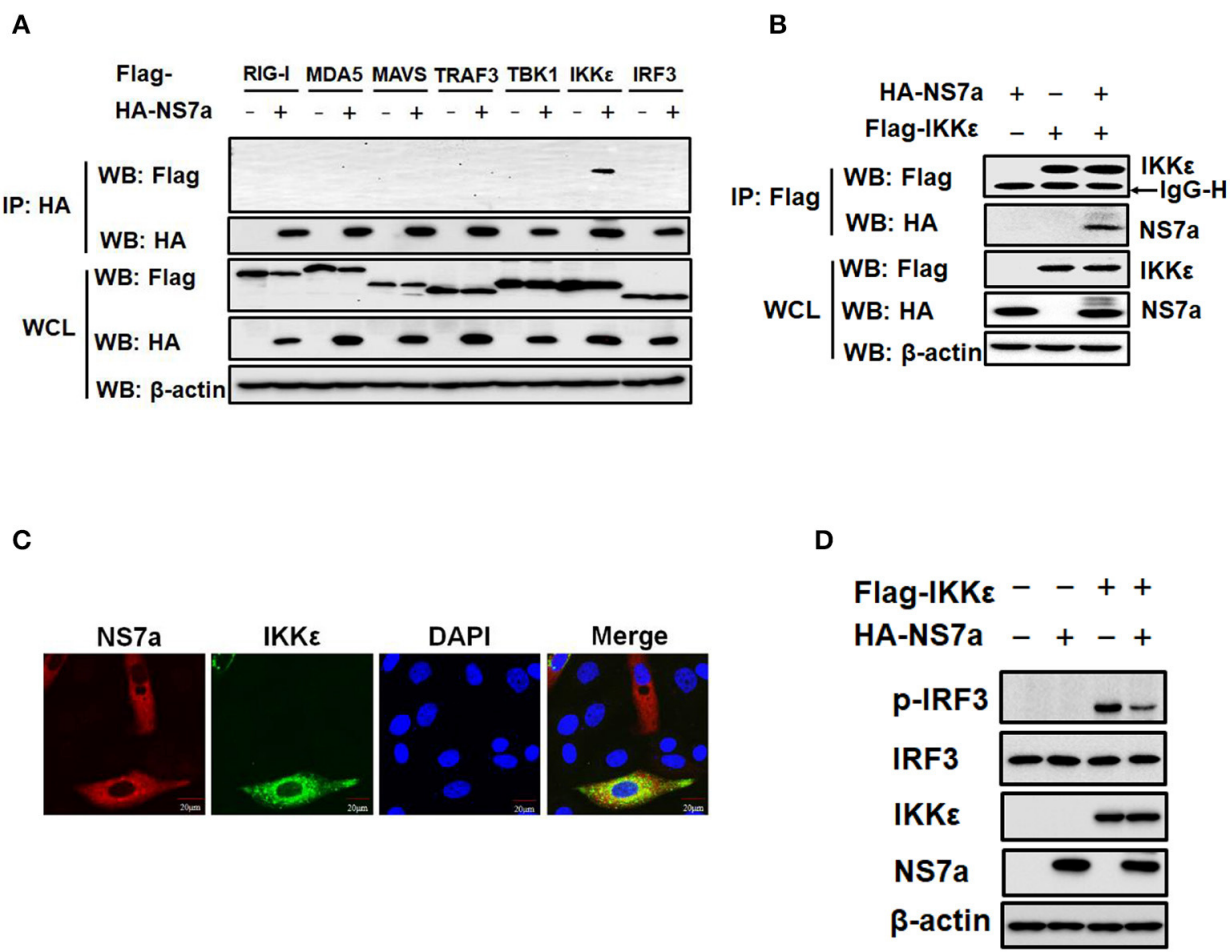
NS7a interacts with IKKε. **(A)** HEK-293T cells were transfected with pCAGGS-HA-NS7a and Flag-tagged RIG-I, MDA5, MAVS, TRAF3, TBK1, IKKε, and IRF3 expression constructs for 24 h. The cells lysates were harvested and used for the co-immunoprecipitation assay with antibodies against HA (IP: HA). Whole-cell lysates (WCL) and immunoprecipitation (IP) complexes were analyzed by western blotting using antibodies against Flag, HA, or β-actin. **(B)** HEK-293T cells were co-transfected with pCAGGS-HA-NS7a and Flag-tagged IKKε expression plasmids. At 24 h post-transfection, the cell lysates were prepared and subjected to immunoprecipitation analysis with anti-Flag (IP: Flag). The WCL and IP complexes were analyzed as described for **(A)**. **(C)** LLC-PK1 cells were co-transfected with pCAGGS-HA-NS7a and Flag-tagged IKKε expression plasmids for 24 h. The cells were fixed and subjected to immunofluorescence analysis to detect NS7a (red) and IKKε (green) using anti-HA and anti-Flag antibodies, respectively. The cell nuclei were stained with DAPI (blue). **(D)** HEK-293T cells were co-transfected with Flag-tagged IKKε expression plasmids and pCAGGS-HA-NS7a or an empty vector for 24 h. The cell lysates were harvested and subjected to western blot analysis using antibodies against anti-p-IRF3 (Ser386), total IRF3, HA, Flag, and β-actin, respectively.

The potential interaction of NS7a with selected components of the RLR signaling pathway was investigated to further identify the molecule(s) targeted by NS7a. Co-IP experiments were performed by co-transfecting pCAGGS-HA-NS7a and Flag-tagged expression plasmids encoding RIG-I, MDA5, MAVS, TRAF3, TBK1/IKKε, or IRF3. The results showed that IKKε was specifically detected in isolated immunocomplexes with the anti-HA MAb but not the closely related TBK1 and other signaling molecules (Figure 4A). In a reverse Co-IP experiment, NS7a was also efficiently co-immunoprecipitated with IKKε via the anti-Flag MAb (Figure 4B). Furthermore, IFAs also demonstrated that Flag-IKKε colocalizes with HA-NS7a, which was distributed in the cytoplasm predominately (Figure 4C). To further test the effect of NS7a on IKKε-mediated IFN-β production, the level of IRF3 phosphorylation induced by IKKε was analyzed in the presence or absence of NS7a. The results showed that IKKε overexpression markedly promoted the level of IRF3 phosphorylation when compared with that of the empty vector-transfected cells. However, the increase was inhibited significantly in the presence of NS7a (Figure 4D). Taken together, these results indicated that NS7a specifically interacts with IKKε but not the closely related TBK1, leading to the inhibition of IKKε function.

### NS7a Competes With TRAF3 and IRF3 for Binding to IKKε

**Figure 5:**
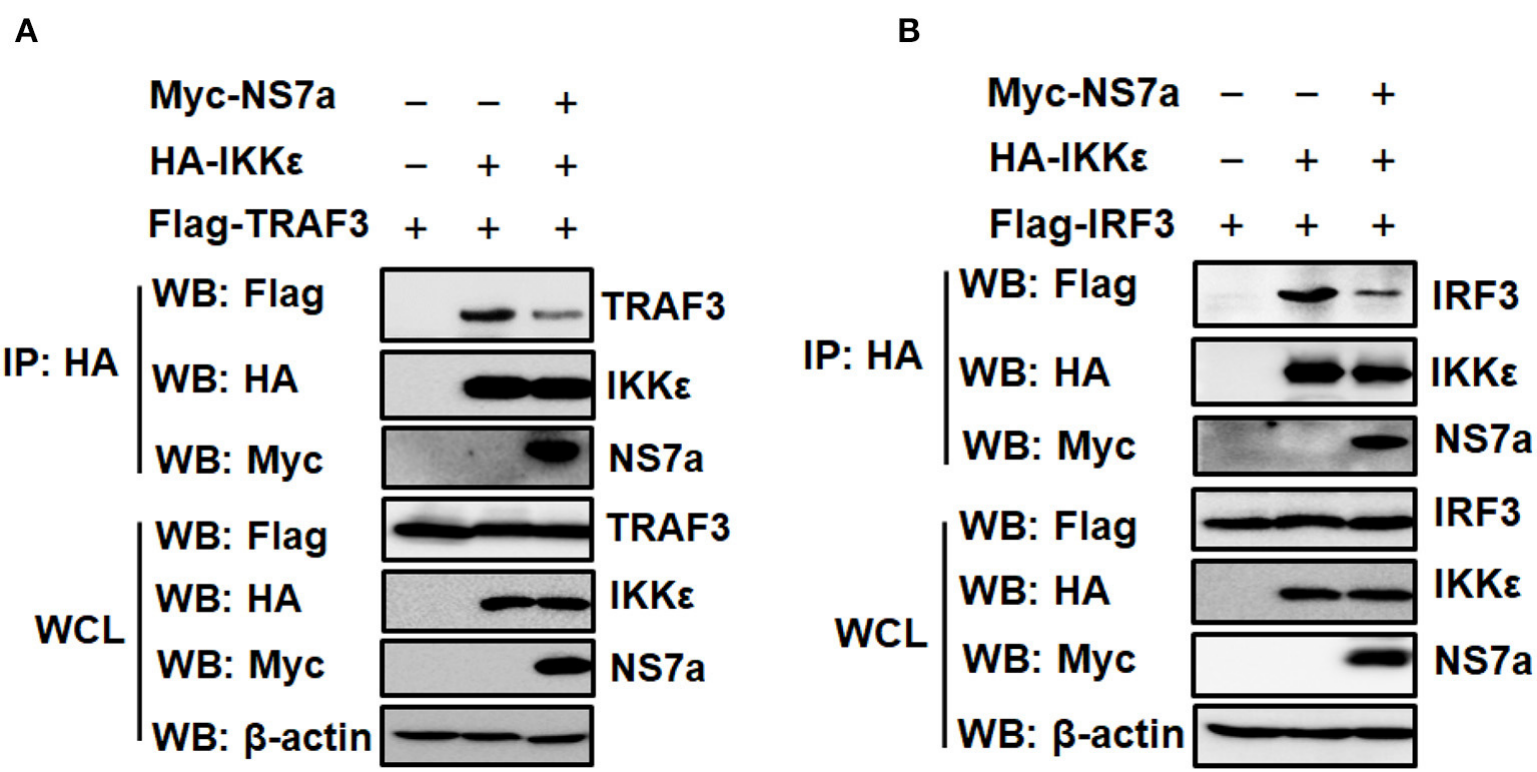
NS7a disrupts the interaction of IKKε with TRAF3 or IRF3. **(A,B)** HEK-293T cells were co-transfected with pCAGGS-HA-IKKε and Flag-tagged TRAF3 **(A)** or Flag-IRF3 **(B)** expression plasmids along with the presence or absence of pCAGGS-Myc-NS7a. At 24 h post-transfection, cells were harvested and lysed. The cell lysates were used for the Co-IP assay with the anti-HA antibody and subsequent western blot analysis using anti-HA, anti-Flag, anti-Myc, and β-actin antibodies. The expression of transfected plasmids was verified in whole cell lysates.

IKKε is a crucial kinase in the RLR pathway that is recruited by the upstream TRAF3 to MAVS; IKKε then recruits downstream transcription factor IRF3, leading to IRF3 activation and subsequent IFN production (Prins et al., 2009; Fang R. et al., 2017). Due to the specific interaction of NS7a with IKKε, we tested whether NS7a is capable of disrupting the interaction of IKKε with its vital upstream signaling component TRAF3 or downstream molecule IRF3. To this end, a competitive Co-IP experiment was performed. HEK-293T cells were co-transfected with HA-tagged IKKε and Flag-tagged TRAF3/IRF3 expression constructs along with pCAGGS-Myc-NS7a or the empty vector. At 24 h post-transfection, the cell lysates were prepared, and subjected to the Co-IP assay with the anti-HA MAb. As shown in Figure 5, TRAF3 (Figure 5A) and IRF3 (Figure 5B) were clearly co-immunoprecipitated by IKKε in the absence of NS7a. However, a significantly lower quantity of TRAF3 or IRF3 in the isolated immunocomplexes was detected in the presence of NS7a when compared with that of the empty vector control (Figures 5A,B).

### NS7a Interacts With KD and SDD of IKKε

**Figure 6:**
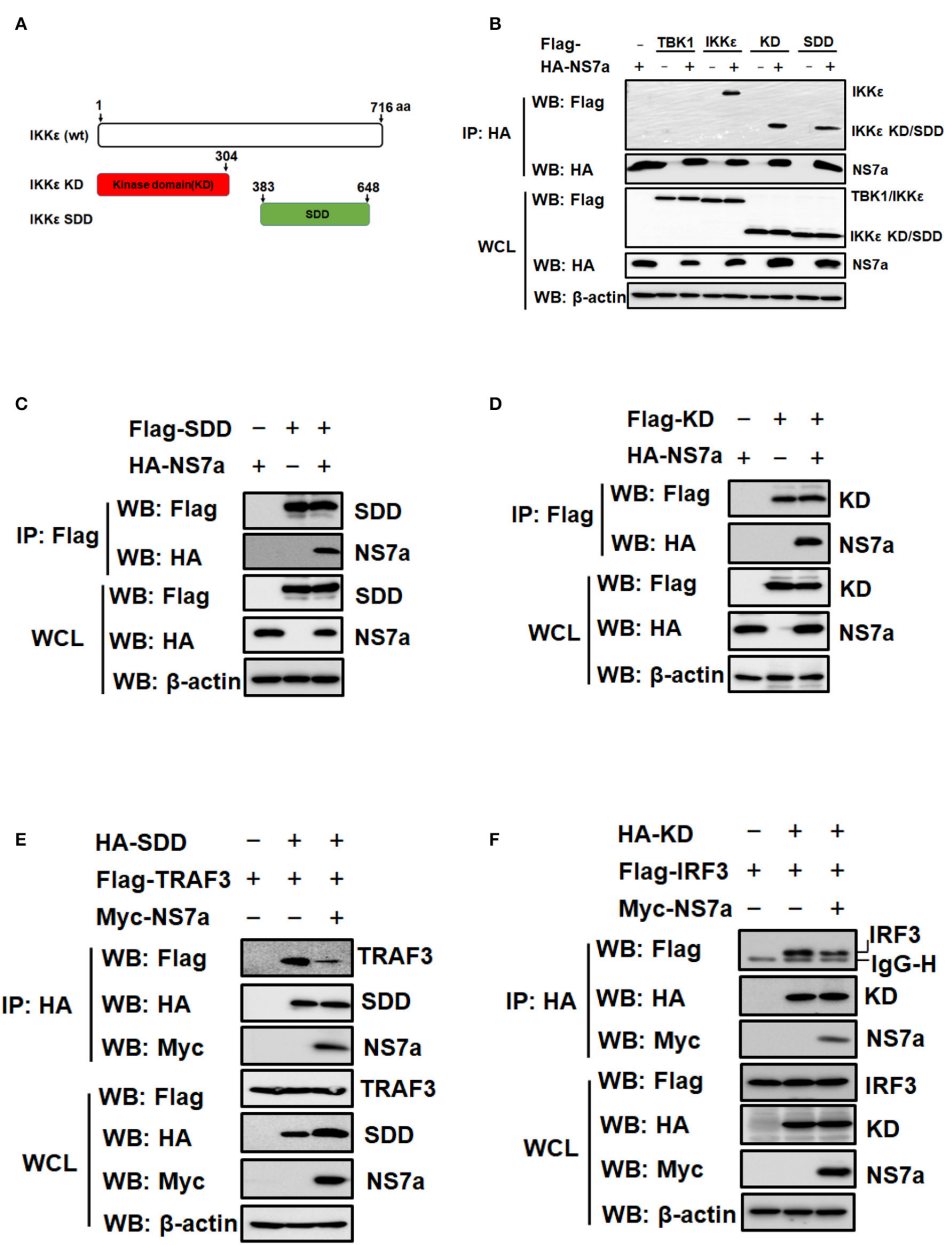
NS7a interacts with both the kinase domain (KD) and the scaffold dimerization domain (SDD) of IKKε. **(A)** Schematic diagram of full-length IKKε (wt) and the truncated protein encoding KD and SDD of IKKε. **(B)** HEK-293T cells were co-transfected with pCAGGS-HA-NS7a and Flag-tagged TBK1, IKKε, KD, or SDD expression plasmids for 24 h, respectively. The Flag-tagged TBK1 was used as a negative control in this experiment. The cell lysates were collected and used for the co-immunoprecipitation assay with the anti-HA antibody. The WCL and IP complexes were analyzed by western blotting using antibodies against Flag, HA, or β-actin. **(C,D)** HEK-293T cells were co-transfected with pCAGGS-HA-NS7a and pCAGGS-Flag-SDD **(C)** or pCAGGS-Flag-KD **(D)** for 24 h, respectively. The Co-IP experiment was performed with anti-Flag as described for **(B)**. **(E,F)** HEK-293T cells were co-transfected with pCAGGS-HA-SDD and Flag-TRAF3 expression plasmids **(E)** or with pCAGGS-HA-KD and Flag-IRF3 **(F)** expression plasmids along with the presence or absence of pCAGGS-Myc-NS7a. At 24 h post-transfection, cells were harvested and subjected to the Co-IP assay with the anti-HA antibody, as described for **(B)**.

IKKε contains two key domains, KD and SDD, which are responsible for interacting with TRAF3 and IRF3, respectively (Prins et al., 2009; Fang R. et al., 2017) (Figure 6A). Because NS7a competes with TRAF3 and IRF3 for binding to IKKε, it is possible that NS7a simultaneously binds to the KD and SDD of IKKε. To confirm this hypothesis, a Co-IP experiment was performed by co-transfecting pCAGGS-HA-NS7a with Flag-tagged full-length IKKε, KD or SDD expression plasmid, respectively. Flag-tagged TBK1 was used as a negative control. The results showed that NS7a co-immunoprecipitated with SDD, KD and full-length IKKε. As expected, no association between NS7a and TBK1 was observed (Figure 6B), and this was in line with the results from Figure 4A. In a reverse Co-IP experiment, SDD and KD also co-immunoprecipitated with NS7a by the anti-Flag MAb efficiently (Figures 6C,D). Competitive Co-IP experiments were performed with the anti-HA MAb to further determine whether NS7a impedes the interaction of TRAF3-SDD and KD-IRF3. The results clearly showed that TRAF3 (Figure 6E) and IRF3 (Figure 6F) are co-immunoprecipitated by SDD and KD in the absence of NS7a, respectively. However, a significantly lower amount of TRAF3 or IRF3 was detected in isolated immunocomplexes in the presence of NS7a when compared with that of the empty vector control. These results indicated that NS7a competes with TRAF3 and IRF3 for binding to the SDD and KD of IKKε, respectively.

### PDCoV Infection Attenuates the Interaction of IKKε With TRAF3 and IRF3

**Figure 7:**
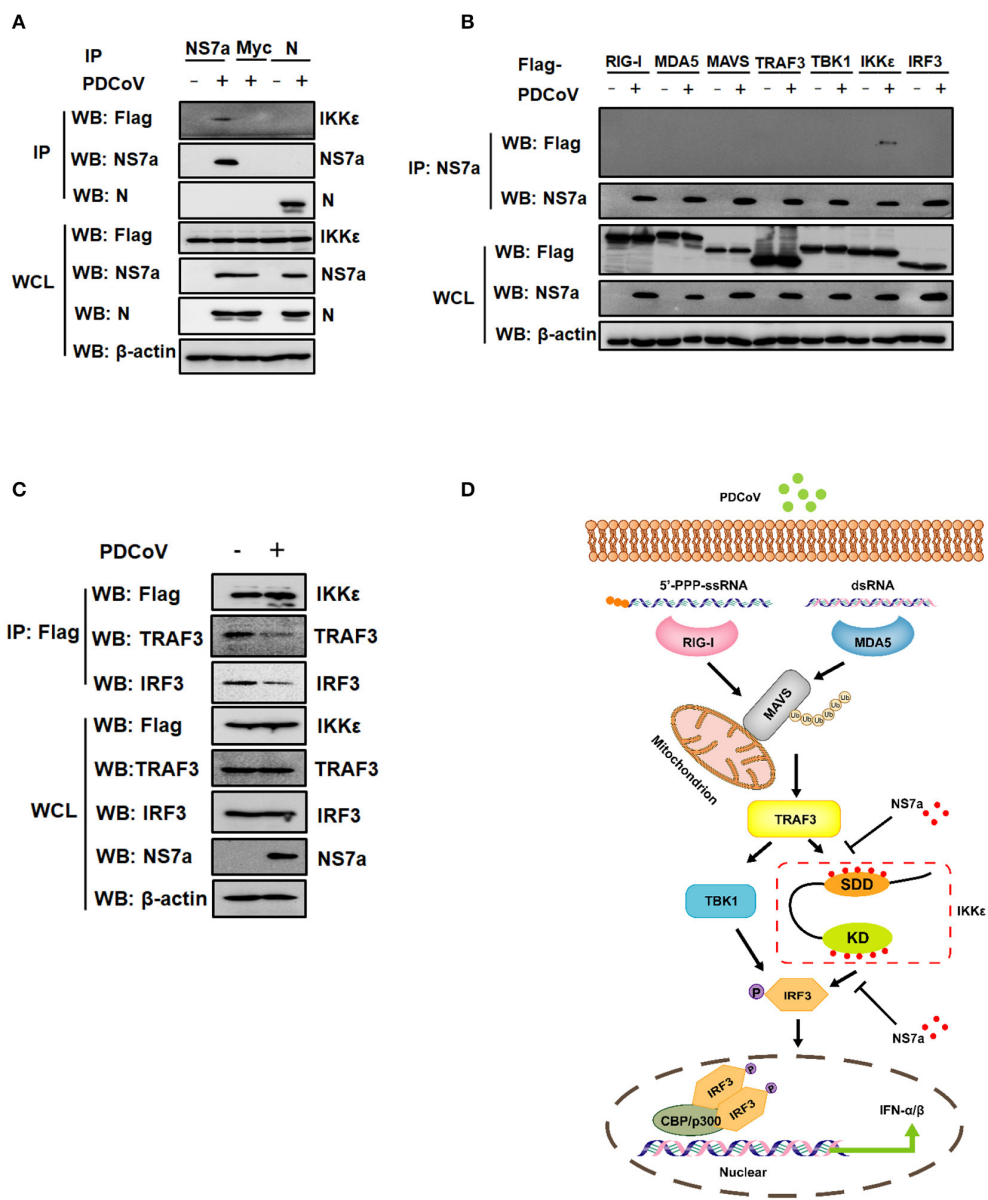
Infection with PDCoV attenuates the interaction of IKKε with TRAF3 and IRF3. **(A)** IPI-2I cells stably expressing Flag-tagged IKKε were infected or mock infected with PDCoV at an MOI of five. After 12 h post-infection, the cell lysates were harvested and subjected to Co-IP analysis using antibodies against PDCoV NS7a, N, or Myc as described for Figure 6B. The WCL and IP complexes were analyzed by western blotting using antibodies against Flag, PDCoV NS7a, N, and β-actin. **(B)** IPI-2I cells were transfected with expression constructs encoding Flag-tagged RIG-I, MDA5, MAVS, TRAF3, TBK1, IKKε, and IRF3 for 12 h and then mock-infected or infected with PDCoV for 12 h. The cells lysates were harvested and used for Co-IP assay with antibodies against NS7a (IP: NS7a). The WCL and IP complexes were analyzed by western blotting using antibodies against Flag, NS7a, or β-actin. **(C)** IPI-2I cells stably expressing IKKε were infected or mock infected with PDCoV at an MOI of five for 12 h. The cell lysates were collected and subjected to Co-IP analysis using the anti-Flag antibody. The WCL and IP complexes were analyzed by western blotting using antibodies against Flag, TRAF3, IRF3, PDCoV NS7a, and β-actin. **(D)** A schematic showing the mechanism of NS7a inhibition of the RLR-mediated signaling pathway. PDCoV NS7a binds to both the SDD and KD of IKKε and competes with TRAF3 and IRF3 for binding to IKKε, leading to the inhibition of upstream and downstream signaling of IKKε.

The results in the overexpression system clearly showed that PDCoV NS7a interacts with IKKε and weakens the interactions of TRAF3-IKKε and IKKε-IRF3. Whether NS7a interacts with IKKε under the conditions of PDCoV infection remains unclear. Because an antibody against endogenic IKKε is ineffective, an IPI-2I cell line stably expressing Flag-tagged IKKε was established by a recombinant lentivirus. As shown in Figure 7A, Flag-tagged IKKε was clearly detected via a Co-IP experiment with a MAb against NS7a rather than a MAb against nucleocapsid (*N*) protein in PDCoV-infected IPI-2I cells stably expressing Flag-IKKε. We also examined whether NS7a interacts with IKKε but not with other components (RIG-I, MDA5, MAVS, TRAF3, TBK1, and IRF3) in PDCoV-infected cells. The results showed that only IKKε was specifically detected in the isolated immunocomplexes with the anti-NS7a MAb but not in other components tested, suggesting that NS7a specifically interacts with IKKε (Figure 7B). We further examined whether the interaction between IKKε and TRAF3 or IRF3 are similarly impaired under PDCoV infection. IPI-2I cells stably expressing Flag-IKKε were infected or mock-infected with PDCoV, and Co-IP experiments were then performed with the anti-Flag antibody. As shown in Figure 7C, the endogenous TRAF3 and IRF3 co-immunoprecipitated by Flag-IKKε were clearly lower in the PDCoV-infected group when compared with that of the mock-infected group. Taken together, these results indicated that PDCoV NS7a at least partially functions to disrupt the binding of IKKε to TRAF3 and IRF3, leading to the inhibition of IFN-β production (Figure 7D).

## Discussion

CoV accessory proteins are species-specific and have been investigated widely over the past decade, and their immune regulation functions, particularly roles in regulating IFN production, and signaling transduction, have been revealed. Moreover, some previously unknown accessory proteins from CoVs have been found in virus-infected cells [e.g., IR ORF from IBV (Bentley et al., 2013), the ORFX encoded by the SARS-like coronavirus (Zeng et al., 2016) and NS7a from PDCoV (Fang P. et al., 2017), highlighting that CoVs possess a larger coding capacity than previously thought]. In this study, we provided evidence that PDCoV NS7a is an IFN antagonist that targets the kinase IKKε, which differs to the mechanism used by the PDCoV accessory protein NS6.

PDCoV NS7a is a unique accessory protein that is translated via an independent subgenomic RNA, and its coding sequence is identical to the C-terminus of NS7 within the *N* gene (Fang P. et al., 2017). There are also several cases describing an alternative ORF encoding accessory protein inside the *N* gene in the genome of beta-CoVs, such as ORF9b of SARS-CoV and ORF I of MHV (Fischer et al., 1997; Meier et al., 2006; Liu et al., 2014). In this study, PDCoV NS7a was found to function as an IFN antagonist independent of cell type, as demonstrated using three different cell lines. Because NS7a is an in-frame, C-terminal peptide of the NS7 protein, we postulated that NS7 might also have IFN antagonistic property. Results from a luciferase reporter assay showed that NS7 also inhibited IFN production induced by SeV, but its antagonistic properties were significantly lower than that of NS7a although the expression level of NS7 was higher than that of NS7a under the same transfection condition (data no shown). We speculated that the N-terminal domain of NS7 may negatively regulate its C-terminal domain structure for antagonizing IFN production. Interestingly, the expression level of NS7a was higher than that of NS7 under the condition of PDCoV infection (Zhang et al., 2020). Based on their differences of expression levels in PDCoV-infected cells and their ability to antagonize IFN, they appear not to be functionally redundant proteins for PDCoV infection. However, a recent report indicated that the replication and pathogenicity of NS7 deletion mutants, which completely abolished both NS7 and NS7a gene expression via changing the initiation codon ATG of NS7 gene and its following seven downstream ATGs to ACGs, resembled those of wild type PDCoV (Zhang et al., 2020). The result indicated the NS7 protein is not critical for PDCoV replication and virulence *in vivo*. This may not fully reflect the role of independent NS7 or NS7a protein on virus replication and pathogenicity but may reflect a combined effect of simultaneous depletion of NS7 and NS7a. Previous studies also indicated that certain accessory proteins encoded by coronaviruses, such as IFN antagonists, can play a role of positive or negative regulation in the virus replication (Cruz et al., 2011; Zhao et al., 2012). For example, the deletion of TGEV ORF7 facilitates the virus replication (Cruz et al., 2011), while the deletion of MHV ns2 significantly attenuates virus replication (Zhao et al., 2012). Certainly, whether various functions of NS7 and NS7a on viral replication contribute to the phenotype of the NS7 deletion mutants need to be confirmed further.

IKKε and TBK1 are two critical kinases in the RLR signaling pathway for type I IFN production. Thus, it is not surprising that many viruses have evolved various mechanisms that target these two kinases to antagonize IFN production. For example, the SARS-CoV M protein binds to multiple key signaling molecules, including RIG-I, TBK1, IKKε, and TRAF3, and disrupts the formation of a complex between TRAF3 and IKKε/TBK1 (Siu et al., 2009). There are also several reports showing that viral proteins inhibit interactions between TBK1 or IKKε and their downstream molecules. For example, Ebola virus protein VP35 interacts with both TBK1 and IKKε, disrupting the interaction of TBK1/IKKε with IRF3 to inhibit type I IFN production (Prins et al., 2009). PEDV N protein and Hepatitis C virus (HCV) NS3 bind to both TBK1 and IKKε, and antagonize IFN-β production by impairing the interaction between TBK1 and IRF3 (Ding et al., 2014). Other viral proteins, like the herpes simplex virus 1 (HSV-1) γ_1_34.5 protein, interact with TBK1 and block its association with downstream IRF3 (Verpooten et al., 2009). However, whether formation of the IKKε-IRF3 complex is blocked by PEDV N, HCV NS3, and HSV-1 γ134.5 remains poorly defined. In this study, we screened several key molecules in the RLR signaling pathway via a Co-IP assay and found that NS7a specifically targets IKKε rather than the closely related TBK-1, which appears to be different from the action pattern found in the abovementioned examples. This finding is unexpected, and the underlying mechanism remains unknown. Indeed, there is a similar report for the arenavirus nucleoprotein, which specifically interacts with IKKε but does not bind to TBK1, leading to the inhibition of IKKε-mediated IFN signaling (Pythoud et al., 2012). IKKε and TBK1 are structurally and enzymatically similar and share over 60% sequence identity. Initial evidence indicated a more important role for TBK1, rather than IKKε, in the induction of type I IFN in response to dsRNA and virus infection (Hemmi et al., 2004; McWhirter et al., 2004). However, follow-up studies demonstrated that IKKε is also critically involved in IFN-β induction, and both IKKε and TBK1 are required for optimal IFN induction upon virus infection, suggesting important non-redundant roles for IKKε, and TBK1 (Pythoud et al., 2012). Furthermore, a previous report showed that virus-inducible IKKε, but not TBK1, is strongly recruited to the mitochondria via MAVS (Lin et al., 2006). The specificity of PDCoV NS7a (this study) and arenavirus nucleoprotein (Pythoud et al., 2012) for IKKε, but not TBK1, are very interesting and deserve further investigation.

The interaction between IKKε and NS7a raises two possibilities that NS7a disrupts the association of IKKε with its upstream adaptors, as observed for the mechanism used by the SARS-CoV M protein that blocks the formation of the TRAF3-TBK1/IKKε complex (Siu et al., 2009), or prevents the recruitment of IKKε to its downstream transcription factors, as observed for the mechanism used by the Ebola virus protein VP35, which interacts with IKKε to inhibit IKKε-IRF3 interaction (Prins et al., 2009). In this study, both TRAF3 and IRF3 coimmunoprecipitated by IKKε decreased notably under the conditions of NS7a overexpression and PDCoV infection. These results suggest that NS7a acts as a competitor of both TRAF3 and IRF3 for IKKε binding, leading to the inhibition of type I IFN production. IKKε kinase contains two key functional domains, SDD and KD, which have been confirmed to mediate the interaction of TRAF3-IKKε and IKKε-IRF3, respectively (Prins et al., 2009; Fang R. et al., 2017). Previous studies also indicated that certain viral proteins interact with IKKε KD to block IKKε-mediated IFN signaling and perturb innate antiviral defense. For example, the lymphocytic choriomeningitis virus (LCMV) nucleoprotein specifically binds to the KD of IKKε, which inhibits IKKε activity to phosphorylate IRF3 (Pythoud et al., 2012). The nonstructural 2B/3 (NS2B/3) protease encoded by the Dengue virus potentially interacts with the IKKε KD and affects its functionality, facilitating inhibition of the IFN signaling response (Anglero-Rodriguez et al., 2014). In addition, the human T-cell leukemia virus type 1 (HTLV-1) HBZ protein, and the *P* protein encoded by rabies virus (RABV) street strains also specifically interact with IKKε and further inhibit IKKε-mediated IRF3 activation; a detail mechanism is, however, missing (Masatani et al., 2016; Narulla et al., 2017). In contrast to the above reported mechanisms, NS7a not only binds to the KD but also the SDD of IKKε, competing with TRAF3 and IRF3 for their binding, respectively. Simultaneous interaction of NS7a with both the SDD and KD of IKKε may afford a more stable complex that effectively impairs IKKε-mediated type I IFN signaling.

As an emerging porcine enteric CoV with potential to cross the species barrier and even its zoonotic potential, PDCoV has received more attention and considerable progress in understanding the biology of PDCoV has been made in the past 5 years (Shang et al., 2018; Xiong et al., 2018; Zheng et al., 2018; Qin et al., 2019; Zhang et al., 2019). Currently, nonstructural proteins nsp5 (Zhu et al., 2017a,b) and nsp15 (Liu et al., 2019), structural nucleocapsid (*N*) protein (Chen et al., 2019; Likai et al., 2019), accessory proteins NS6 (Fang et al., 2018), and NS7a (this study) encoded by PDCoV have been identified as IFN antagonists. However, it is noteworthy that several studies indicated that PDCoV induces type I IFN production in infected piglets. For example, Jung et al. detected serum cytokine responses of gnotobiotic pigs with acute PDCoV infection and found that serum IFN-α and IL-22 were increased at 1 day post-inoculation (dpi) (Jung et al., 2018); Xu et al. reported that inoculating conventional weaned piglets with PDCoV at a high dose (1 × 10^9^ TCID_50_/head) by oral feeding could induce mRNA expression of TLR3, IL-12, IFN-α, IFN-β, and PKR in Peyer's patches at 3 dpi, but not at 7 dpi, indicating that PDCoV infection induces innate immune responses during the early infection *in vivo*, while it overcomes the antiviral innate immunity to infect the body at the late infection (Xu et al., 2019). In our previous study, we found that PDCoV infection did not induced IFN-β expression at 24 h post-infection and even suppressed SeV-induced IFN-β production in cell cultures (Luo et al., 2016). Indeed, in PDCoV-infected cells (LLC-PK1), low levels of mRNA expression of IFN-β could also be detected. Similar observations were also reported for other CoVs, such as PEDV, SARS-CoV, and MERS-CoV (Chen et al., 2010; Annamalai et al., 2015; Mahallawi et al., 2018). For example, PEDV infection in suckling pigs leads to the increased serum IFN-α in the early stages (Annamalai et al., 2015); In the early period of infection, a modest increase in the level of IFN-α and IFN-β can be detected in the lungs of SARS-CoV-infected BALB/c mice (Chen et al., 2010). There is also an increase of plasma IFN-α2 in MERS-CoV-infected patients (Mahallawi et al., 2018). The different results obtained from studies *in vivo* and *in vitro* need to be investigated further. It is possible that the dsRNA replication intermediates generated during virus replication contribute to the induction of low or moderate level of IFN production before the expression of virus-encoded IFN antagonists, such as PDCoV NS7a in this study. The immune system of the body is certainly more complex than that of cell culture system. Other unidentified factors may contribute to the differences observed *in vivo* and *in vitro* during virus infection.

## Data Availability Statement

The datasets generated for this study are available on request to the corresponding author.

## Author Contributions

PF, LF, and SXiao conceived and designed research. PF, SXia, JR, JZ, and DB performed the experiments. PF and YZ analyzed the data. PF wrote the manuscript with critical input from LF, GP, SZ, and SXiao. All authors discussed results and contributed to manuscript preparation.

## Conflict of Interest

The authors declare that the research was conducted in the absence of any commercial or financial relationships that could be construed as a potential conflict of interest.

## Supplementary Material

Supplementary Tables Raw luciferase data (Excel).
